# Neonatal outcome in macrosomia and gestational diabetes mellitus depending on the definition of macrosomia: a retrospective cohort study

**DOI:** 10.1186/s12884-026-09840-5

**Published:** 2026-08-25

**Authors:** Luisa Laura Meurer, Sabine Körber, Johannes Stubert

**Affiliations:** Department of Obstetrics and Gynecology, Rostock University Medical Center, Suedring 81, 18059 Rostock, Germany

**Keywords:** Macrosomia, Large for gestational age, Gestational diabetes mellitus, Neonatal outcome, Umbilical artery, Pulsatility index

## Abstract

**Introduction:**

Gestational diabetes increases the risk of neonatal macrosomia, which is associated with maternal and neonatal morbidity. However, the definition of macrosomia is inconsistent and includes both a fixed weight limit and a gestational age-dependent percentile. This study aimed to evaluate the association between different definitions of macrosomia and short-term neonatal outcome in pregnancies complicated by gestational diabetes.

**Material and methods:**

In this retrospective single-center cohort study, 882 women with gestational diabetes and a singleton pregnancy were included. Adverse neonatal outcome was defined as 5-min Apgar score below eight and/or admission to the neonatal intensive care unit. We compared three different definitions of macrosomia: 1.) birthweight ≥ 4000 g, 2.) birthweight > 90th percentile, and 3.) length-related birthweight > 90th percentile. Eutrophic newborns served as controls and small-for-gestational-age infants were excluded. Additionally, umbilical artery Doppler parameters were explored.

**Results:**

Overall, 220 out of 882 newborns (24.9%) met at least one definition of macrosomia: 129 (14.6%) with birthweight ≥ 4000 g, 126 (14.3%) with birthweight > 90th percentile, and 205 (23.2%) with length-related birthweight > 90th percentile. Short-term adverse neonatal outcome occurred in 6.2% (*n* = 55). In the total cohort, macrosomia was not associated with adverse outcome. Newborns with a birthweight > 90th percentile showed a higher, but not statistically significant, rate of adverse outcome (odds ratio (OR) 1.5 [0.8–3.1], *p* = 0.231). In analyses restricted to macrosomic infants, those with birthweight > 90th percentile had higher rates of adverse outcome compared with those ≤ 90th percentile (8.7% vs. 1.1%; OR 8.9, 95% CI 1.1–70.2). This remained associated after adjustment for preterm birth (adjusted OR 8.8, 95% CI 1.1–71.4), although preterm birth was the strongest overall predictor. The effect was mainly observed in infants weighing < 4000 g. Umbilical artery Doppler showed limited predictive value.

**Conclusions:**

Macrosomia represents a heterogeneous group not uniformly associated with adverse short-term neonatal outcome. Within this cohort, birthweight > 90th percentile, particularly in infants < 4000 g, was associated with higher rates of adverse outcome, whereas fixed weight thresholds were not. Gestational age–adjusted definitions may better identify at-risk neonates.

**Supplementary Information:**

The online version contains supplementary material available at 10.1186/s12884-026-09840-5.

## Key Message

The definition of macrosomia is inconsistent. We compared the adverse neonatal outcome for three different definitions. In contrast to birthweight ≥ 4000 g and length-related birthweight > 90th percentile, occurrence of adverse outcome was increased only in birthweight > 90th percentile.

**Supplementary Information:**

The online version contains supplementary material available at 10.1186/s12884-026-09840-5.

## Introduction

The birth of a macrosomic neonate is associated with health risks for both the mother and the newborn [1]. Gestational diabetes mellitus (GDM) increases the risk of macrosomia, as well as adverse maternal and/or neonatal outcomes [2]. The link between increased glucose supply to the fetus due to elevated maternal blood glucose levels and subsequent excessive fetal growth is well documented [3, 4]. Non-glucose related factors such as impaired lipid metabolism appear to contribute to the development of macrosomia [5]. In addition to obstetric complications directly attributable to fetal disproportion, such as an increased risk of caesarean section, vaginal-operative deliveries, shoulder dystocia or birth injuries of higher degree[6–9], neonatal adverse outcome such as low Apgar scores, meconium aspiration, respiratory distress, hypoglycemia are common reasons for a stay in the neonatal intensive care unit (NICU) [10–12].

Even in the long term, especially in the presence of GDM, there is an increased risk of developing obesity or type 2 diabetes mellitus [11, 13, 14].

Despite these known associations, there is no clear definition of neonatal macrosomia [1, 15]. While some use a birthweight cut-off of 4000 g or even 4500 g as the threshold, others refer to a birthweight > 90th or 95th percentile (= large for gestational age, LGA) [1, 15].

Neonatal macrosomia resulting from GDM is characterized by an increase of fat and not lean body mass [5]. Although not yet commonly used, the length-related birth weight (g/cm) > 90th percentile could also be useful for the definition of macrosomia, analogous to the body mass index (BMI, kg/m^2^), which is the basis of the WHO definition of obesity in adults [16].

While the primary aim of this study is to evaluate postnatal neonatal risk according to different definitions of macrosomia, we additionally explored umbilical artery Doppler parameters as a potential antenatal correlate of fetal overgrowth because altered placental hemodynamics have been linked to fetal growth patterns. This exploratory analysis was intended to assess whether commonly available prenatal parameters may contribute to risk stratification beyond birthweight-based definitions.

## Methods

### Study design and participants

Of 1104 women with GDM who delivered singletons ≥ 34 weeks' gestation at our tertiary care center from 2020 to 2021, 882 were included in this retrospective cohort analysis. The GDM diagnosis was based on a 75 g oral glucose tolerance test in accordance to the IADPSG guideline [17].

**Fig. 1:**
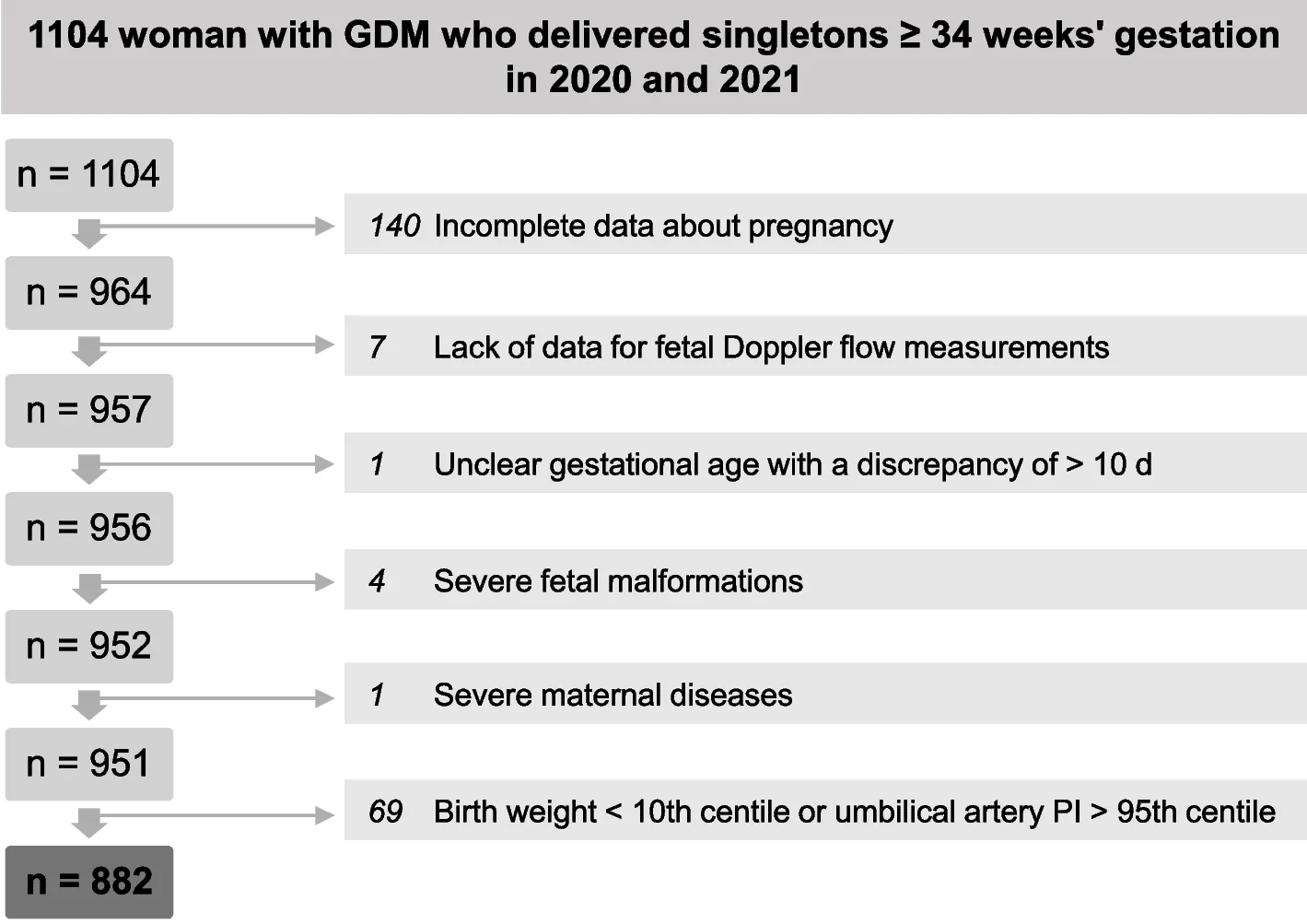
Flowchart of the inclusion and exclusion criteria of the patient collective

Criteria for exclusion were lack of data for pregnancy and birth including fetal Doppler flow measurements, unclear gestational age with a discrepancy of more than 10 days for estimated date of delivery, fetal malformations, and severe maternal diseases (e.g. renal insufficiency ≥ stage III, heart failure ≥ NYHA III). We also excluded cases with a neonatal birthweight < 10th percentile (small for gestational age, SGA) or an increased resistance of the umbilical arteries with a pulsatility index (PI) > 95th percentile, as this is associated with placental insufficiency and fetal growth restriction (Fig. 1) [16, 18]. The last Doppler measurement was used for analysis. Doppler examination was part of routine clinical care at our outpatient clinic.

### Data collection

Maternal characteristics recorded included age at delivery, pre-pregnancy weight and weight gain during pregnancy, nicotine abuse during pregnancy, gravidity, parity, insulin requirement for GDM, presence of hypertensive pregnancy disorders and gestational age at delivery. Fetal parameters recorded were sex, birthweight and length-related birthweight as well as their percentiles and height of the newborn.

Birthweight percentiles were calculated based on German reference charts according to Voigt et al. [16] Short-term adverse neonatal outcome was defined as requirement of a neonatal intensive care unit (NICU) stay and/or a 5-min Apgar score below eight. We compared the following definitions of neonatal macrosomia: 1) birthweight > 90th percentile, 2) length-based birthweight > 90th percentile and 3) birthweight ≥ 4000 g. The control group consisted of the remaining eutrophic children who did not meet any of these criteria.

The composite outcome was chosen to reflect clinically relevant early neonatal compromise in a retrospective setting, where more granular morbidity data (e.g., hypoglycemia, respiratory morbidity, or birth trauma) were not consistently available.

### Statistical analysis

Data were stored and analyzed using the statistical package IBM SPSS 20 (SPSS Inc. Chicago, IL, USA), Excel 2021 (Microsoft Corporation, Redmond, WA, USA) and the statistic software R 4.4.1 with RStudio (version 2024.09.0). Descriptive statistics included mean and standard deviation (SD) for parametric parameters and median and interquartile range (IQR) for non-parametric parameters. For categorical variables, the frequency and relative percentage were reported. To test for differences, we used the unpaired t-test with Welch correction or the Mann–Whitney U test for continuous variables and the Chi-square test or Fisher's exact test for categorical variables. All *p-*values were obtained using two-sided statistical tests, and values < 0.05 were considered statistically significant. We determined the risk of the subgroups with regard to the occurrence of a neonatal adverse outcome using odds ratios (OR) including 95% confidence intervals (95% CI). To account for multiple testing, *p-*values were adjusted using the Holm–Bonferroni procedure, controlling the family-wise error rate at α = 0.05.

We determined the adjusted ORs using logistic regression analysis.

Variable selection for multivariable models was based on a combination of clinical relevance and strength of association in univariable analysis rather than purely automated procedures. The number of outcome events per variable was considered to minimize overfitting and was consistent with commonly recommended thresholds.

A total of 55 outcome events were available, resulting in an events-per-variable ratio of approximately 18 in the primary multivariable model.

Variables were entered sequentially according to the magnitude of their crude odds ratios, beginning with the strongest association. In the adjusted models for the prediction of adverse outcome, several covariates were included. Variables not improving the model performance were excluded. Collinearity among predictors was assessed using variance inflation factors (VIFs). All VIFs were below commonly accepted thresholds (VIF < 2), indicating no relevant multicollinearity. Model calibration was assessed using the calibration slope obtained by regressing the observed outcome on the predicted log odds. Model calibration was acceptable, with calibration slopes close to unity across all models (see supplement). For description of the relationship between umbilical artery PI and birthweight a correlation analysis according to Pearson with specification of the correlation coefficient (ρ) and a linear regression analysis with specification of the coefficient of determination (R^2^) was performed. Receiver operating characteristics (ROC) curves and the area under the curve (AUC) were computed, and the optimal cut-off value (minimal distance to sensitivity and specificity of 1) was calculated using the following equation: (1-sensitivity)^2^ + (1-specificity)^2^.

Predictive performance of birthweight > 90th percentile versus macrosomia was evaluated using category-based NRI (risk thresholds 5% and 10%) and IDI based on logistic regression–derived predicted probabilities. IDI confidence intervals were obtained by bootstrap resampling (2000 iterations) using the nricens package in R. No a priori sample size calculation was performed due to the retrospective study design.

## Results

### Characteristics of the study population according to macrosomia definition

Among the 882 pregnancies, 14.3% (*n* = 126) had a neonatal birthweight > 90th percentile, 23.2% (*n* = 205) had a length-related birthweight > 90th percentile, and 14.6% (*n* = 129) had a birthweight ≥ 4000 g.

**Fig. 2:**
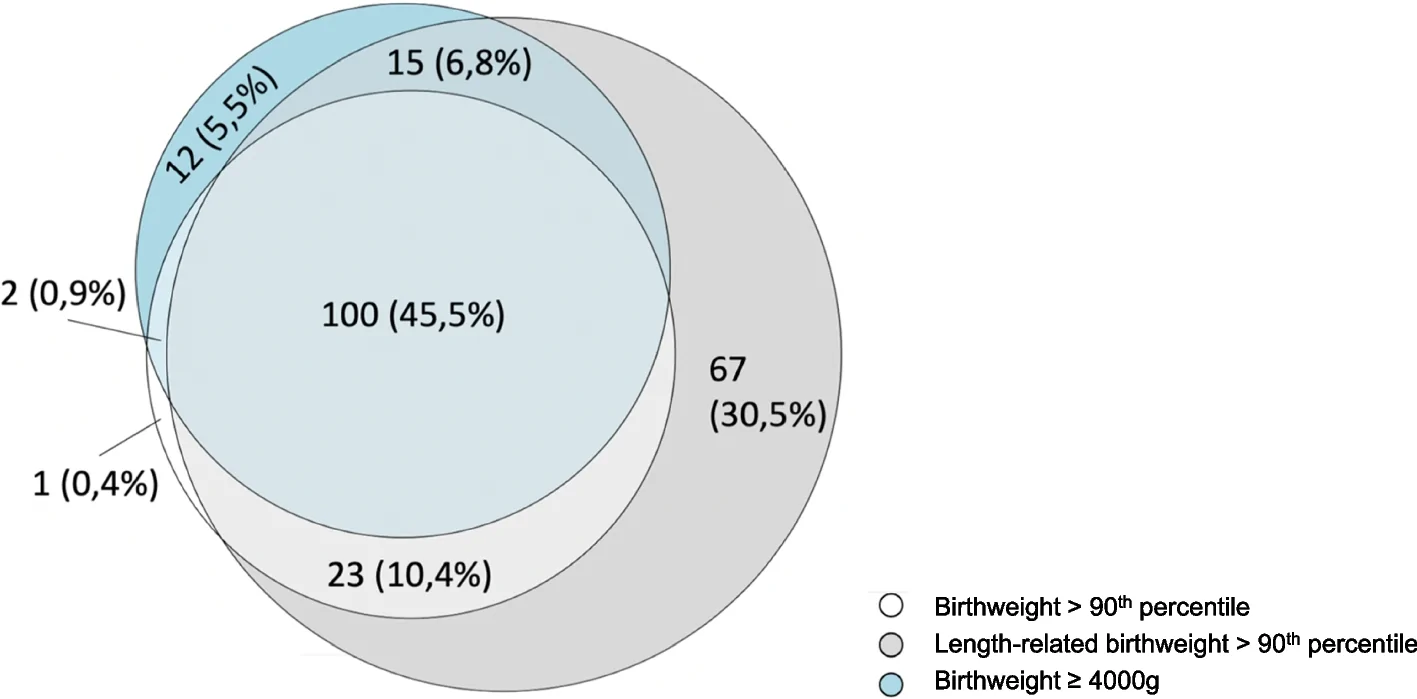
Venn diagram of macrosomia definitions. Absolute and relative frequencies within the subgroups are indicated

Duplications within the groups were allowed if cases could be assigned to more than one group (Fig. 2). Overall, 220 newborns (24.9%) met at least one definition of macrosomia. One hundred newborns (11.3% of the total population and 45.5% of macrosomic infants) fulfilled all three criteria. Eighty newborns (9.1% of the total cohort and 36.4% of macrosomic infants) met only one criterion. Among these, infants with a birthweight > 90th length-related percentile (*n* = 67) constituted the largest subgroup (Fig. 2). The majority of newborns (75.1%, *n* = 662) were eutrophic. Maternal and neonatal characteristics of the different subgroups are summarized in Table 1.

**Table 1:** Maternal and neonatal characteristics depending on the definition of macrosomia

	All (*n* = 882)	Eutrophic (*n* = 662)	Birthweight > 90th percentile (*n* = 126)	*p-*value*	Length-related birthweight > 90th percentile (*n* = 205)	*p-* value**	Birthweight ≥ 4000 g (*n* = 129)	*p-*value***
Maternal age, years (mean ± SD)	32.4 ± 5.1	32.6 ± 5.1	31.6 ± 5.0	0.053	31.8 ± 5.0	0.062	31.5 ± 4.9	0.017
BMI before pregnancy, kg/m^2^ (mean ± SD)	29.4 ± 6.9	28.8 ± 6.7	31.4 ± 7.1	< 0.001	31.5 ± 7.1	< 0.001	31.4 ± 7.1	< 0.001
Weight gain during pregnancy (median, IQR)	12 (7–16)	11 (7–16)	13 (9–17)	0.025	13 (7–17)	0.128	14 (9–17)	0.002
Smoking during pregnancy, n (%)	59 (6.7%)	47 (7.1%)	7 (5.6%)	0.7	12 (5.9%)	0.164	7 (5.4%)	0.572
Gravidity, n (median, min–max)	2 (1–16)	2 (1–16)	2 (1–10)	0.084	2 (1–10)	0.047	2 (1–10)	0.272
Parity, n (median, min–max)	2 (1–10)	2 (1–10)	2 (1–8)	0.014	2 (1–8)	0.006	2 (1–6)	0.096
Primiparity, n (%)	331 (37.5%)	269 (40.6%)	34 (27.0%)	0.004	58 (28.3%)	0.002	38 (29.5%)	0.018
Umbilical PI < 25. percentile, n (%)	188 (21.3%)	126 (19.0%)	45 (35.7%)	< 0.001	61 (29.8%)	0.002	39 (30.2%)	0.006
Insulin-requiring gestational diabetes, n (%)	399 (45.2%)	257 (38.8%)	91 (72.2%)	< 0.001	138 (63.4%)	< 0.001	85 (65.9%)	< 0.001
Hypertensive diseases, n (%)	113 (12.8%)	83 (12.5%)	18 (14.3)	0.34	28 (13.7%)	0.72	15 (11.6%)	0.884
Gestational age at delivery, days (median, IQR)	275 (269–281)	275 (268–281)	274 (269–279)	0.429	274 (270–281)	0.903	277 (273–282)	0.001
Preterm birth < 37 weeks, n (%)	40 (4.5%)	37 (5.6%)	3 (2.4%)	0.093	4 (2.0%)	0.019	0 (0%)	0.001
Male newborns, n (%)	449 (50.9%)	347 (52.4%)	55 (43.7%)	0.08	89 (43.4%)	0.025	70 (54.3%)	0.773
Birthweight, g (mean ± SD)	3542 ± 441	3372 ± 344	4184 ± 252	< 0.001	4050 ± 284	< 0.001	4226 ± 193	< 0.001
Birthweight percentile (median, IQR)	59 (36–81)	49 (30–65)	96 (93–98)	< 0.001	92 (85–97)	< 0.001	95 (92–98)	< 0.001
Height, cm (mean ± SD)	50.2 ± 2.0	49.8 ± 1.9	52.1 ± 1.5	< 0.001	51.3 ± 1.8	< 0.001	52.2 ± 1.4	< 0.001
Length-related birthweight, g/cm (mean ± SD)	70.4 ± 7.1	67.6 ± 5.4	80.5 ± 4.1	< 0.001	79.1 ± 4.0	< 0.001	80.8 ± 3.7	< 0.001
Length-related birthweight percentile (median, IQR)	73 (49–90)	61 (40–78)	98 (97–99)	< 0.001	97 (94–99)	< 0.001	98 (95–99)	< 0.001
NICU stay, n (%)	34 (3.9%)	26 (3.9)	7 (5.6%)	0.464	8 (3.9%)	1	6 (4.7%)	0.631
5’-APGAR < 8, n (%)	25 (2.8%)	19 (2.9%)	6 (4.8%)	0.268	6 (2.9%)	1	2 (1.6%)	0.555
Neonatal adverse outcome, n (%)	55 (6.2%)	43 (6.5%)	11 (8.7%)	0.34	12 (5.9%)	0.87	6 (4.7%)	0.55

^*^ birthweight > 90th percentile vs eutrophic newborns

^**^ length-related birthweight > 90th percentile vs eutrophic newborns

^***^ birthweight ≥ 4000 g vs eutrophic newborns

Compared with eutrophic newborns, all three macrosomia definitions were associated with higher pre-pregnancy maternal body mass index (BMI) and more frequent insulin therapy. Maternal weight gain during pregnancy was higher in cases with birthweight > 90th percentile and ≥ 4000 g.

Mothers of macrosomic newborns were older and more frequently multiparous. The frequency of preterm birth was lower in newborns with a length related birthweight > 90th percentile and/or ≥ 4000 g, but not among infants with a birthweight > 90th percentile. Moreover, neonates with a length-related birthweight > 90th percentile were more frequently female.

### Umbilical artery pulsatility index and birthweight

The pulsatility index (PI) of the umbilical artery was inversely associated with birthweight even after exclusion of SGA newborns with a correlation coefficient of ρ = −0.259 (*p* < 0,001) and a coefficient of determination R^2^ = 0.067 (Figure S1). ROC-analysis yielded an AUC of 0.60 (0.54–0.65, *p* = 0.001), with an optimal cut-off at PI < 23.5th percentile for predicting birthweight > 90th percentile. However, diagnostic performance at a clinically applicable cut-off of the 25th percentile was limited (sensitivity 35%, specificity 82%, positive predictive value 23%, positive likelihood ratio 1.9, negative likelihood ratio—0.8). Accordingly, umbilical artery resistance does not appear clinically suitable for predicting macrosomia. Furthermore, umbilical artery PI was not predictive of adverse outcome among macrosomic neonates (ROC-AUC 0.58 [95% CI 0.40–0.76; *p* = 0.395]).

### Short-term adverse neonatal outcomes

The combined endpoint occurred in 6.2% of all newborns (*n* = 55). No overall association was observed between macrosomia and adverse outcome (Table 1). Infants with birthweight > 90th percentile (*n* = 126) showed a higher, but not significant, rate of adverse outcome compared with the remaining 756 neonates (8.7% vs. 5.8%, *p* = 0.231, OR 1.54 [95% CI 0.8–3.1]). Nevertheless, of all 55 newborns with adverse outcome, only 20% had a birthweight > 90th percentile.

Newborns with a length-related birthweight > 90th percentile (5.9% vs. 6.4%, *p* = 0.87, OR 0.92 [95% CI 0.5–1.8]) and those weighing ≥ 4000 g (4.7% vs. 6.5%, *p* = 0.555, OR 0.7 [95% CI 0.3–1.7]) showed no increased risk.

### Analysis restricted to macrosomic neonates

**Fig. 3:**
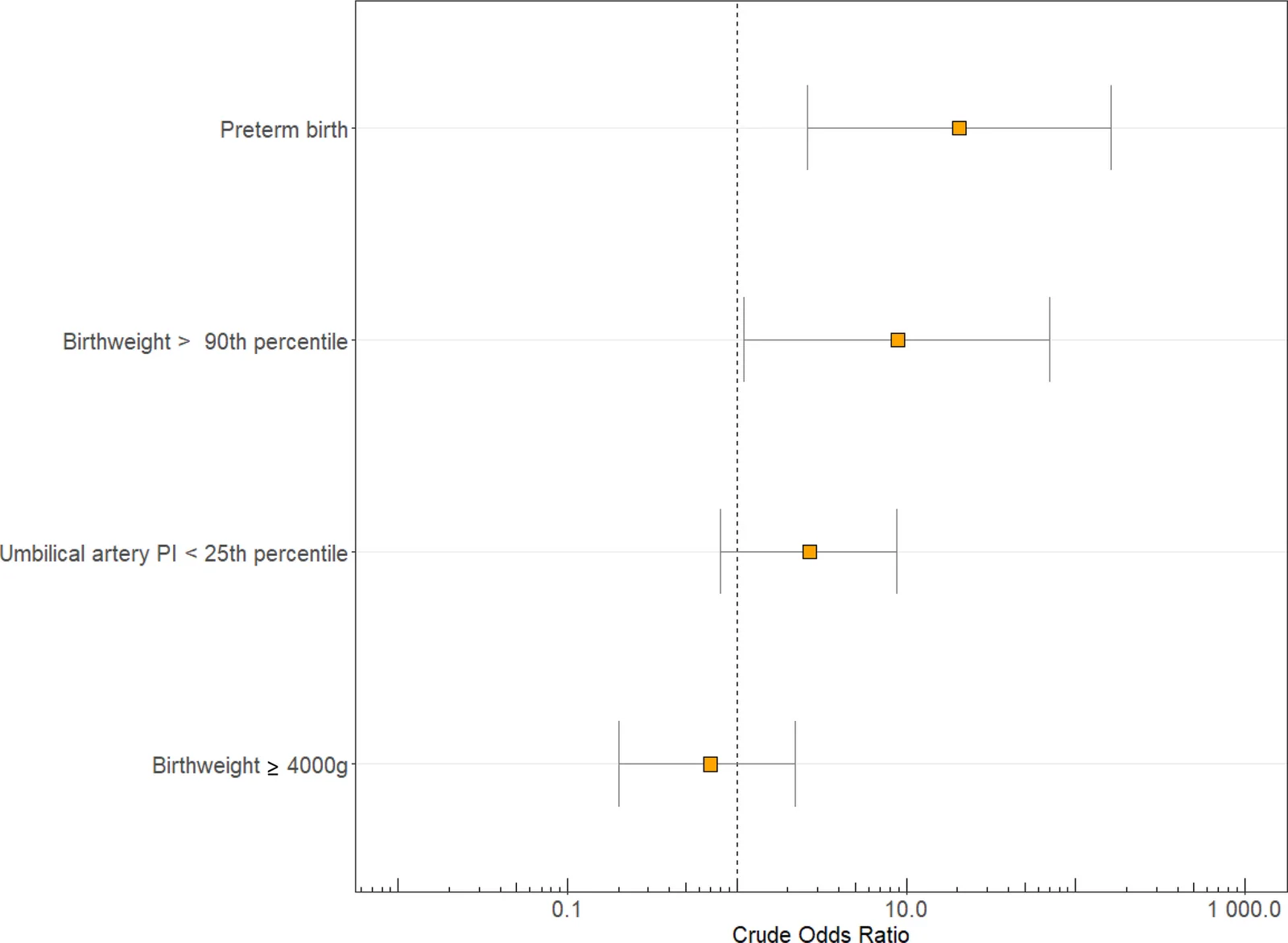
Risk factors for adverse neonatal outcomes in macrosomic neonates. Odds ratios with 95% CI are shown for the cohort of neonates with at least one positive criterion of macrosomia

Among infants meeting *at least one macrosomia criterion* (*n* = 220), adverse outcome was more common in those with a birthweight > 90th percentile (*n* = 126) compared to those ≤ 90th percentile (*n* = 94; 8.7% vs. 1.1%; *p* = 0.015; OR 8.9 [95% CI 1.1–70.2]) (Table 2, Fig. 3, Table S1).

**Table 2:** Putative risk factors for neonatal adverse outcome (AO). Odds ratios (ORs) are presented for a) the total cohort (upper part) and b) the subgroup of newborns with at least one criterion of macrosomia (lower part)

Risk factor for AO	% with AO	% without AO	crude OR (95% CI)	*p-*value*
*a) Total cohort*				
Preterm birth	34.5	2.7	19.3 (9.6–38.8)	< 0.001
Hypertensive disorder during pregnancy	25.5	12.0	2.5 (1.3–4.8)	0.010
Birthweight > 90th centile	20.0	13.9	1.5 (0.8–3.1)	0.231
Maternal obesity (BMI ≥ 30 kg/m^2^)	47.3	42.0	1.2 (0.7–2.1)	0.482
Birthweight > 4000 g	10.9	14.9	0.7 (0.3–1.7)	0.555
Macrosomia (at least one criterion positive)	21.8	25.2	0.8 (0.4–1.6)	0.633
Primiparity	34.5	37.8	0.9 (0.5–1.5)	0.669
Insulin treatment	41.8	45.5	0.9 (0.5–1.5)	0.675
Male gender	49.1	51.1	0.9 (0.5–1.6)	0.783
Macrosomia (all criteria positive)	9.1	11.5	0.8 (0.3–2.0)	0.825
Length-related birthweight > 90th centile	21.8	23.4	0.9 (0.5–1.8)	0.870
PI of umbilical artery < 25th centile	20.0	21.4	0.9 (0.5–1.8)	1.000
*b) Subgroup with at least one criterion of macrosomia*				
Preterm birth	16.7	1.0	20.6 (2.6–161.7)	0.015
Birthweight > 90th centile	91.7	55.3	8.9 (1.1–70.2)	0.015
PI of umbilical artery < 25th centile	50.0	26.9	2.7 (0.8–8.8)	0.102
Primiparity	58.3	72.6	1.9 (0.6–6.2)	0.326
Maternal obesity (BMI ≥ 30 kg/m^2^)	66.7	52.4	1.8 (0.5–6.2)	0.386
Male gender	33.3	47.1	0.6 (0.2–1.9)	0.390
Birthweight > 4000 g	50.0	59.1	0.7 (0.2–2.2)	0.559
Hypertensive disorder during pregnancy	16.7	13.5	1.3 (0.3–6.2)	0.670
Insulin treatment	66.7	64.4	1.1 (0.3–3.8)	1.000
Macrosomia (all criteria positive)	41.7	45.7	0.9 (0.3–2.8)	1.000
Length-related birthweight > 90th centile	100.0	92.8		1.000

^*^ Exact test to Fisher

This association was primarily driven by infants weighing < 4000 g despite being > 90th percentile (19.0%, *n* = 24/126). In this subgroup, adverse outcome occurred in 25.0% vs. 0.0% in the remaining macrosomic infants (*p* < 0.001). The median gestational age at delivery was 38 weeks (interquartile range 37–38 weeks) and 12.5% (*n* = 3/24) were delivered preterm < 37 weeks.

We conducted a logistic regression analysis and ascertained that the observed effect was strongly attributable to preterm birth (adjusted OR 19.9 [95% CI 2.1–186.1]). Nevertheless, birthweight > 90th percentile remained associated with adverse outcome even after adjustment (adjusted OR 8.8 [95% CI 1.1–71.4]) (Table 3).

**Table 3:** Adjusted odds ratios (OR) of risk factors for neonatal adverse outcome. a) for the total cohort (upper part) and b) for the subgroup of newborns with at least one criterion of macrosomia (lower part)

Risk factor for AO	Adjusted OR	95% CI	*p-*value
*a) Total cohort*			
Preterm birth	19.8	9.63–40.74	< 0.001
Hypertensive disorder during pregnancy	2.1	1.02–4.26	0.043
Birthweight > 90th percentile	2.0	0.96–4.25	0.064
*b) Subgroup with at least one criterion of macrosomia*			
* Model 1*			
Preterm birth	19.9	2.13–186.09	0.009
Birthweight > 90th percentile	8.8	1.08–71.41	0.043
* Model 2*			
Preterm birth	24.5	2.87–208.84	0.003
PI of umbilical artery < 25th percentile	3.0	0.88–10.49	0.078
* Model 3*			
Preterm birth	22.5	2.26–224.58	0.008
Birthweight > 90th percentile	7.4	0.90–61.32	0.063
PI of umbilical artery < 25th percentile	2.3	0.65–8.07	0.200

Results of the sensitivity analysis restricted to term births were consistent with the main analysis, with similar effect directions and magnitudes (OR 7.3 [95% CI 1.3–135.2]). This finding suggests that the observed association is not solely explained by prematurity. This analysis reduces the confounding effect of preterm birth and therefore strengthens the robustness of the observed association.

In contrast, newborns who solely fulfilled the criterion of a length-related birthweight > 90th percentile (*n* = 67) even showed a better outcome in the total cohort (0% vs. 6.7%, *p* = 0.017) as well as in the subgroup with at least one macrosomia criterion (0% vs. 7.8%, *p* = 0.020). Newborns with solely a length-related birthweight > 90th percentile were more common female (62.7% vs. 48.0%, *p* = 0.021), heavier (mean birthweight 3792 ± 165 g vs. 3521 ± 450 g, *p* = 0.007), but not longer (50 ± 1 cm vs 50 ± 2 cm, *p* = 0.74).

Table 2 presents a list of putative risk factors for short term neonatal adverse outcome. In the total cohort only preterm birth < 37 weeks and hypertensive disorder during pregnancy were associated with an increased risk. The strength of the correlation between preterm birth and adverse outcomes was found to be fair (Spearman’s ρ = 0.37, *p* < 0.001).

Primiparity, male gender, low vascular resistance in the umbilical artery, pregravid maternal obesity and insulin treatment of GDM were all not associated with adverse outcome.

After adjustment preterm birth remained the main risk factor regardless of the cohort analyzed, whereas birthweight > 90th percentile remained a significant risk factor only in the subgroup of macrosomic neonates (Table 3).

Collinearity diagnostics showed no relevant multicollinearity among predictors. Model calibration was acceptable, with calibration slopes close to unity across all models. Model discrimination was acceptable, with AUCs ranging from 0.67 to 0.76. The highest discrimination was observed for the model including preterm birth, birthweight > 90th percentile, and umbilical artery PI < 25th percentile (AUC = 0.76, 95% CI 0.63–0.90) (Tables S3 and S4).

#### Reclassification analyses

In the subgroup of macrosomic infants (*n* = 220), category-based NRI analysis using thresholds of 5% and 10% demonstrated substantial upward reclassification among cases (41.7%), indicating improved identification of pregnancies with adverse outcome when using birthweight > 90th percentile as predictor instead of birthweight ≥ 4000 g.

However, upward reclassification among controls was also substantial (46.6%) with only partial compensatory downward reclassification (32.2%).

Event-NRI was positive (NRI + = 0.42, 95% CI 0.10 to 0.73), whereas non-event-NRI was negative (NRI − = − 0.14, 95% CI − 0.27 to − 0.02), yielding a non-significant overall NRI of 0.27 (95% CI − 0.05 to 0.59).

The risk discrimination was further evaluated using Integrated Discrimination Improvement (IDI). Compared with the macrosomia definition, the birthweight > 90th percentile definition significantly improved overall discrimination (IDI = 0.026, 95% CI 0.011–0.038), indicating better separation between pregnancies with and without adverse neonatal outcome.

## Discussion

The Pedersen hypothesis proposes that fetal hypertrophy or macrosomia results from maternal hyperglycemia stimulating fetal insulin production [4]. LGA birthweight is associated with increased obstetric and neonatal risks, particularly mechanically mediated complications such as shoulder dystocia, severe perineal trauma, prolonged labor, postpartum hemorrhage, and caesarean delivery. [1]

However, our findings suggest that increased birthweight alone does not uniformly translate into higher neonatal morbidity in pregnancies complicated by GDM. Both fixed thresholds (≥ 4000 g) and percentile-based definitions include constitutionally large infants without pathological overgrowth. Previous large cohort studies have similarly shown that higher thresholds may better identify neonates at risk of adverse outcome. In an analysis based on a US birth cohort of nearly 18 million newborns, the cut-off for identifying LGA with at least a 2-fold risk of a low 5-min Apgar score at birth was the 97th percentile [19]. Similarly, in a Chinese cohort study, a birthweight of 4500 g or more was found to be better predictive of adverse outcome than 4000 g [20].

In our cohort, macrosomia represented a heterogeneous group. Notably, neonates with birthweight > 90th percentile but < 4000 g showed the highest rates of short-term adverse outcome, whereas infants ≥ 4000 g or with length-related birthweight > 90th percentile did not. Within this cohort, a gestational age–adjusted definition identified a subgroup with higher observed rates of adverse outcome. Importantly, the consistency of results in term births suggests that the observed association cannot be fully attributed to prematurity alone. Moreover, the association persisted after adjustment for preterm birth, although preterm delivery remained the strongest overall predictor.

Unmeasured factors such as maternal characteristics, glycemic control, or obstetric management may have contributed to these findings and residual confounding cannot be excluded.

Shoulder dystocia is a key complication of fetal macrosomia but could not be assessed due to lack of systematic data. Therefore, our results should not be interpreted as contradicting the established association between increasing birthweight and mechanical delivery complications.

The observed heterogeneity underscores the need to distinguish constitutionally large from pathologically overgrown infants, similar to the differentiation between SGA and fetal growth restriction [21]. Low flow resistance in the umbilical artery is associated with the occurrence of fetal macrosomia [22–24]. In this context, we explored umbilical artery Doppler as a potential antenatal correlate. This analysis was exploratory. Although umbilical artery pulsatility index was statistically associated with birthweight, its predictive performance was insufficient for clinical application. Other proposed prenatal markers, such as abdominal wall thickness, have also shown limited utility [25].

This study has several limitations. Its retrospective design introduces potential residual confounding. Relevant variables such as ethnicity, delivery mode, and labor induction were not available. The absence of these variables as well as outcomes like hypoglycemia or birth trauma may have introduced bias and led to underestimation or misclassification of neonatal morbidity. As labor induction may influence both gestational age and neonatal outcome, its absence may have introduced additional bias.

The composite outcome (5-min Apgar score < 8 and/or NICU admission) reflects early neonatal compromise but is heterogeneous. In particular, NICU admission is influenced by institutional practice and may not exclusively indicate severe morbidity. The individual components of the composite outcome differ in clinical severity, which may affect interpretability of the combined endpoint. However, it serves as a pragmatic surrogate in retrospective analyses. Important outcomes such as neonatal hypoglycemia, respiratory morbidity, birth trauma, cesarean section, and operative vaginal delivery were not systematically available. Maternal complications were not assessed. Furthermore, the use of German reference charts may limit generalizability to other populations. In subgroup analyses restricted to macrosomic neonates, the number of outcome events was limited, which may have reduced the stability of the estimates and contributed to the wide confidence intervals. Generally, wide confidence intervals indicate limited precision and should be interpreted with caution.

## Conclusion

In this retrospective cohort of 882 pregnancies complicated by gestational diabetes, macrosomia represented a heterogeneous group that was not uniformly associated with adverse short-term neonatal outcome. Within this cohort, birthweight > 90th percentile, particularly in infants weighing < 4000 g, was associated with higher rates of adverse outcome, whereas fixed weight thresholds (≥ 4000 g) and length-related definitions were not. These findings suggest that gestational age–adjusted definitions may better identify neonates at increased risk, although this observation requires validation in other populations. These findings should be interpreted as descriptive and associative rather than predictive, given the retrospective study design. Clinical decisions, including the timing of delivery or induction in suspected macrosomia, should be made cautiously. Further research is needed to distinguish constitutionally large from pathologically overgrown infants and to improve prenatal risk stratification.

## Supplementary Information

Supplementary Material 1.

Supplementary Material 2.

## Data Availability

Data are available from the corresponding author JS upon reasonable request.

## Abbreviations

AO: Adverse outcome
GDM: Gestational diabetes mellitus
NICU: Neonatal intensive care unit
BMI: Body Mass Index
PI: Pulsatility index
SD: Standard deviation
IQR: Interquartile range
OR: Odds ratio
Adj. OR: Adjusted odds ratio
95% CI: 95% Confidence intervals
SGA: Small for gestational age
AGA: Appropriate for gestational age
LGA: Large for gestational age
FGR: Fetal growth restriction
CTG: Cardiotocography
