# Supplementary material for: Neonatal outcome in macrosomia and gestational diabetes mellitus depending on the definition of macrosomia: a retrospective cohort study
Source: BMC Pregnancy Childbirth. 2026 Aug 25;26:946. doi: 10.1186/s12884-026-09840-5 (PMC13504804; doi:10.1186/s12884-026-09840-5)
Supplement: Supplementary file 1 [file 12884_2026_9840_MOESM2_ESM.docx]

# STROBE Checklist

Study type: Retrospective single-center cohort study
Title: Neonatal Outcomes in Macrosomia and Gestational Diabetes Mellitus According to Different Definitions of Macrosomia

| Item | STROBE Recommendation | Addressed in Manuscript |
| --- | --- | --- |
| 1a | Indicate study design in title/abstract | Retrospective cohort design stated in Abstract and Methods |
| 2 | Scientific background and rationale | Introduction describes pathophysiology and heterogeneity of definitions |
| 3 | Objectives | Clearly stated primary objective |
| 4 | Study design | Retrospective cohort design described early in Methods |
| 5 | Setting | Tertiary care center, 2020–2021, Rostock University Medical Center |
| 6 | Participants | Eligibility and exclusion criteria detailed |
| 7 | Variables | Macrosomia definitions, composite neonatal outcome, covariates defined |
| 8 | Data sources/measurement | Clinical records and Doppler data described |
| 9 | Bias | Adjustment for confounders; discussion of residual confounding |
| 10 | Study size | All eligible cases included; no formal power calculation |
| 11 | Quantitative variables | Percentiles and 4000 g threshold defined |
| 12 | Statistical methods | Regression, ROC, NRI, IDI described |
| 13 | Participants flow | Flow diagram provided (Figure 1) |
| 14 | Descriptive data | Baseline characteristics in Table 1 |
| 15 | Outcome data | 55/882 adverse outcomes reported |
| 16 | Main results | Unadjusted and adjusted ORs reported |
| 17 | Other analyses | Subgroup, ROC, reclassification analyses |
| 18 | Key results summary | First paragraph of Discussion |
| 19 | Limitations | Retrospective design, residual confounding, limited outcome definition |
| 20 | Interpretation | Results interpreted cautiously in context of literature |
| 21 | Generalisability | Tertiary care setting discussed |
| 22 | Funding | None declared |
